# Supplementary material for: The Importance of Charge Transfer and Solvent Screening in the Interactions of Backbones and Functional Groups in Amino Acid Residues and Nucleotides
Source: Int J Mol Sci. 2022 Nov 4;23(21):13514. doi: 10.3390/ijms232113514 (PMC9654426; doi:10.3390/ijms232113514)
Supplement: Supplementary file 1 [file ijms-23-13514-s001.zip › ijms-2000129-supplementary.pdf]

Supporting Information for
The importance of charge transfer and solvent screening in the interactions of backbones and functional groups in amino acid residues and nucleotides

Vladimir Sladek[*,†] and Dmitri G. Fedorov[*,‡]

†*Institute of Chemistry, Slovak Academy of Sciences, Dubravska cesta 9, 845 38 Bratislava, Slovakia*

‡*Research Center for Computational Design of Advanced Functional Materials (CD-FMat) National Institute of Advanced Industrial Science and Technology (AIST), Japan*

E-mail: sladek.vladimir@savba.sk; d.g.fedorov@aist.go.jp

Definition of FMO fragments

In FMO, fragments differ from conventional units by a few shifted atoms, as shown in detail in Figure S1. The red part of Figure S1a shows a conventional nucleotide, and the corresponding red part in Figure S1b shows an FMO fragment. It can be seen that the two units have the same chemical composition; however, FMO fragment k contains the phosphate group of nucleotide $k+1$, whereas its own phosphate is assigned to fragment $k-1$.

Likewise, amino acid residues have the same composition as FMO fragments, but the carbonyl of residue k is assigned to fragment $k+1$, whereas the carbonyl of residue $k-1$ is assigned to fragment k. For both nucleotides and amino acid residues, the backbone definition is shifted, but side chains are the same.

Segments, on the other hand, are identical to conventional nucleotides and amino acid residues.

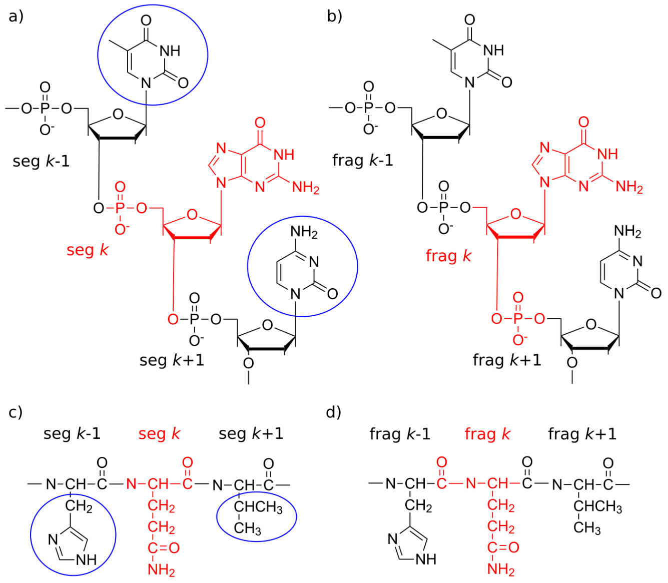

Figure S1: (a) Conventional vs (b) FMO nucleotides; (c) conventional vs (d) FMO amino acid residues. Functional units are in blue circles. Atoms forming one segment or fragment are shown in red.

Structures of important pairs used in the analysis

The structures of all pairs, for which interactions are presented in the main text, are shown in Figure S2.

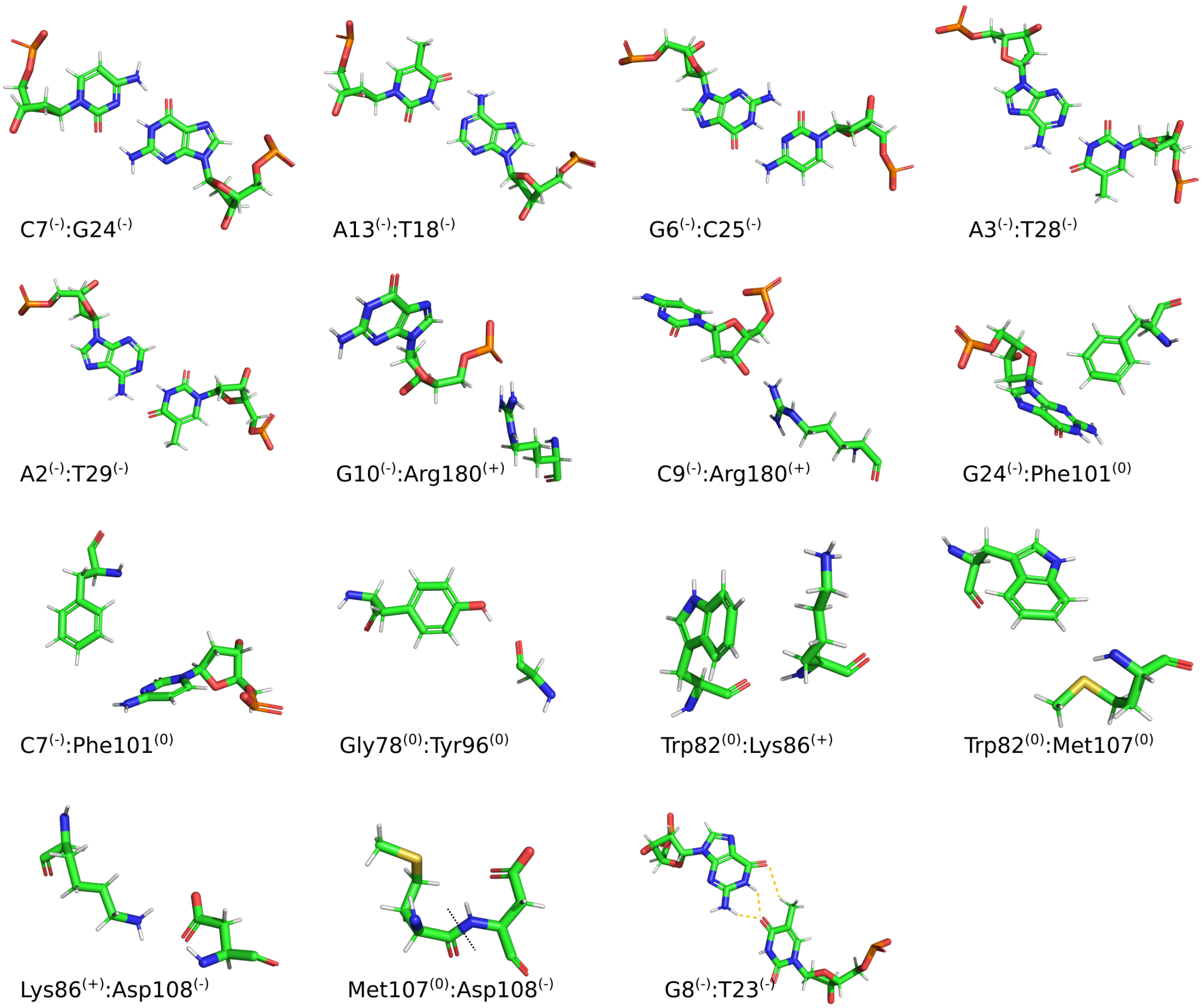

Figure S2: Representative pairs of nucleotides and amino acid residues in the protein-DNA complex (PDB: 2o8b). Formal charges are indicated as superscripts.

In Figure S3, small segments (consisting of 2-3 atoms) are shown, which are used to describe interactions between functional groups.

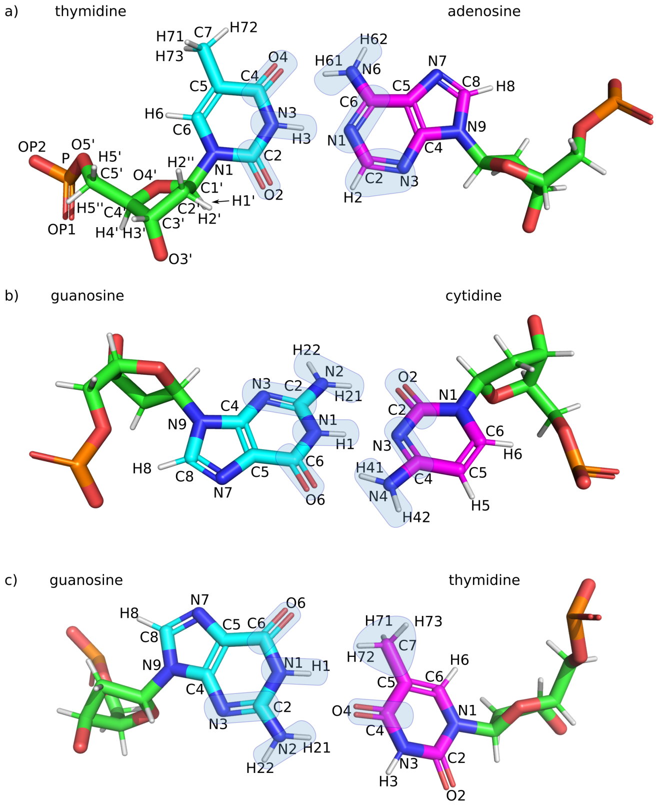

Figure S3: Three types of base pairs, adenine-thymine (A13:T18, regular), cytosine-guanine (C7:G24, regular), and guanine-thymine (G8:T23, mismatch). The shadows define small segments used for an evaluation of interactions between functional groups. Atom types are shown to explain labels used in Figure S4.

Hydrogen bonding analysis

In Figure S4, statistical details on the hydrogen bond lengths are shown. It can be seen that there is a single distinct maximum in most of them, except that in C7:G24 and G8:T23 there are two maxima, possibly related to the competition of two binding poses.

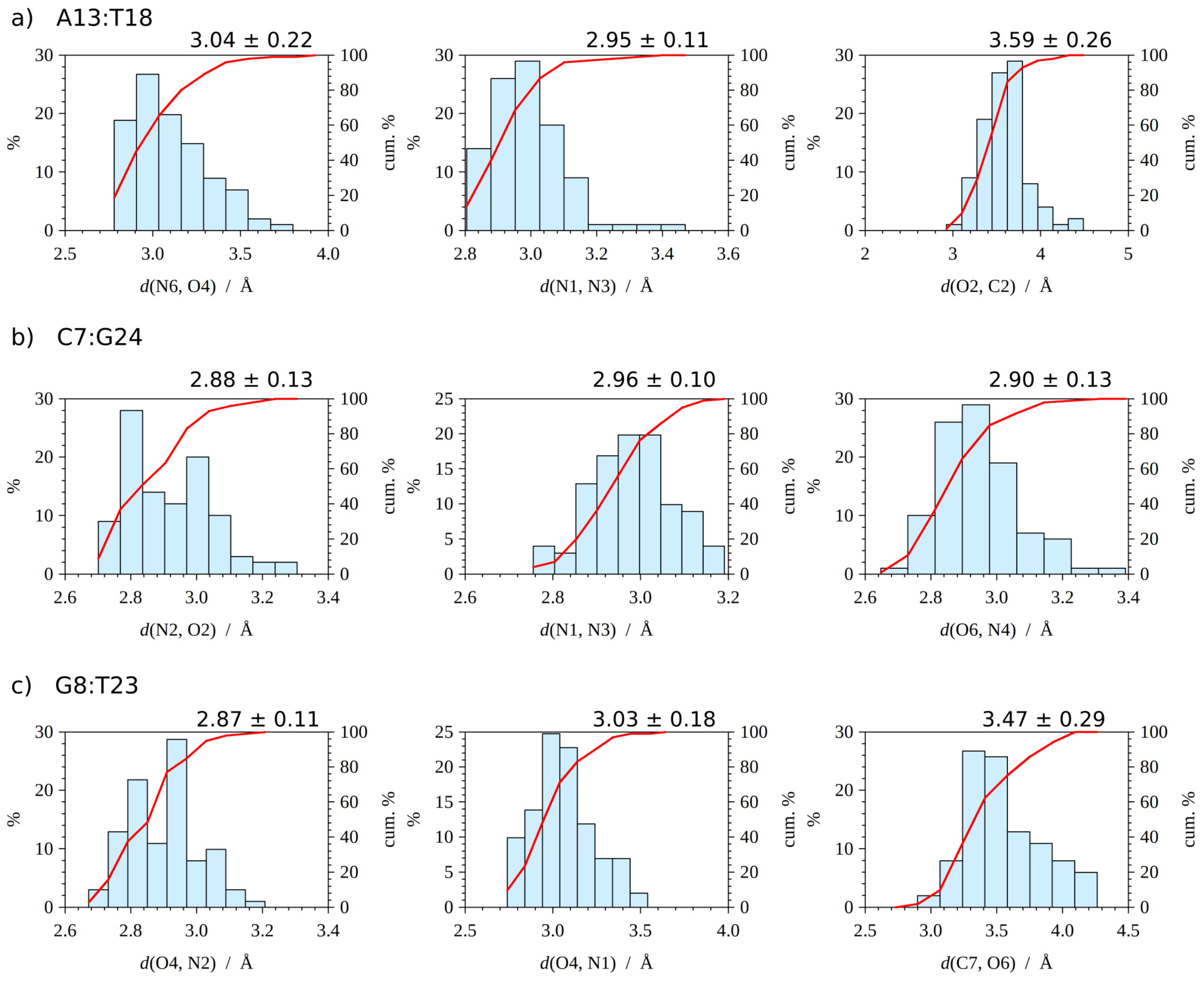

Figure S4: Histograms (blue bars), cumulative histograms (red line) and averaged values of hydrogen bond lengths in the protein-DNA complex (PDB: 2o8b) obtained from 100 MD snapshots.

Statistical analysis and implications to PRN models

Some of the basics of mutual information are described here for an easier explanation of its use.[1,2] For two variables x and y let us determine how much the values of one of them can help to predict the value of the other. For simplicity assume that each variable can take values from a discrete set of n states so that $x \in \{s_k^{(x)}\}_{k=1}^n$ and $y \in \{s_l^{(y)}\}_{l=1}^n$. Let $p_k^{(x)}$ be the probability of x falling into given state k. For a sufficiently large number of measurements N, these form a statistical ensemble for the variable and $p_k^{(x)} = N_k/N$. N_k is the number of measurements when x does belong to state k, $\sum_k N_k = N$, and hence $\sum_k p_k^{(x)} = 1$.

Let $H^{(x)} = -\sum_k p_k^{(x)} \log_2 p_k^{(x)}$ be the entropy of the ensemble of x. A base 2 logarithm originates from the information theory root of the entropy, albeit the interpretation is identical to entropy in statistical physics (apart from the Boltzmann factor kT). The units of entropy are bits here. The larger the entropy value, the higher is the uncertainty of guessing the correct state (because the probability distribution is more uniform). In the information theory, the entropy is the average number of bits one ought to use to encode the states of the variable.

A two-dimensional joint probability distribution $p_{kl}^{(x,y)}$ can be defined for state (s_k^x, s_l^y) denoting the simultaneous occurrence of variable x in state k and variable y in state l. In this case $p^{(x)}$ and $p^{(y)}$ are so called marginal distributions for the two respective variables, obeying $p_k^{(x)} = \sum_l p_{kl}^{(x,y)}$ and $p_l^{(y)} = \sum_k p_{kl}^{(x,y)}$. The entropy $H^{(y)}$ can be defined for y and a joint entropy for the joint probability distribution $H^{(x,y)}$.

$$\begin{aligned} H^{(x)} + H^{(y)} - H^{(x,y)} &= -\sum_k p_k^{(x)} \log_2 p_k^{(x)} - \sum_l p_l^{(y)} \log_2 p_l^{(y)} + \sum_{k,l} p_{kl}^{(x,y)} \log_2 p_{kl}^{(x,y)} = \\ &= -\sum_{k,l} p_{kl}^{(x,y)} \left(\log_2 p_k^{(x)} + \log_2 p_l^{(y)} \right) + \sum_{k,l} p_{kl}^{(x,y)} \log_2 p_{kl}^{(x,y)} = \\ &= \sum_{k,l} p_{kl}^{(x,y)} \log_2 \frac{p_{kl}^{(x,y)}}{p_k^{(x)} p_l^{(y)}} \end{aligned} \tag{1}$$

A relation of the entropies of the individual variables x and y and the entropy when both x and y are considered jointly can be defined. If x and y are completely independent (uncorrelated), then $p_{kl}^{(x,y)} = p_k^{(x)} p_l^{(y)}$. In such case $H^{(x,y)} = H^{(x)} + H^{(y)}$, and the result of eq 1 is zero. Hence eq 1 can express the reduction of entropy for variables x and y if they are not independent compared to the case if they were independent. In other words, if they are correlated, then they share some information. The mutual information (entropy) of variables x and y is defined as

$$MI^{(x;y)} = \sum_{k,l} p_{kl}^{(x,y)} \log_2 \frac{p_{kl}^{(x,y)}}{p_k^{(x)} p_l^{(y)}} \tag{2}$$

In information theory, MI defines the reduction in bits (information) one can potentially save if encoding one variable while knowing the states of the other variable. Hence it is "a portion of the information the two variables share". For each joint state (s_k^x, s_l^y), if there is a positive correlation between x being in state k while y is in state l, $p_{kl}^{(x,y)} > p_k^{(x)} p_l^{(y)}$ and such state contributes positively to MI. In other words, the information content of the state (s_k^x, s_l^y) is greater than the information content of the states k and l individually. MI is measured in bits. It would be more convenient to define a dimensionless relative variable to relate MI and the entropies of x and y. The conditional coefficients of constraint are defined as

$$\begin{aligned} C^{(x|y)} &= \frac{MI^{(x;y)}}{H^{(y)}} \\ C^{(y|x)} &= \frac{MI^{(x;y)}}{H^{(x)}} \end{aligned} \tag{3}$$

Given the entropy $H^{(y)}$ of y, $C^{(x|y)}$ indicates what fraction of the states in x can be predicted. These coefficients are conceptually similar to conditional probability.

In this paper, two graphs $G^{(1)}$ and $G^{(2)}$ are compared, where each of the graphs represents a set of pair interactions. The vertices in the graphs are residues, the edges represent interactions (ΔE_{ij}). A threshold value is used to define edges between vertices (residues),

i.e. if $\Delta E_{ij} \leq E_{\text{lim}}$ then there is an edge between vertices i, j in G and the weight of the edge is ΔE_{ij}, otherwise the edge weight is 0 (disconnected vertices). The edges in the two graphs are considered as the two variables, and each graph contains N^2 "measurements" ($N = 201$ is the number of nodes in the graph). In order to use MI, a set of n discrete states should be obtained.

This is done with histograms. Histogram bins are of equal width, with the bin edge width of $\Delta = R/n$, where R is the energy span the edges have (the edge weights). Since the upper boundary is always 0 and the lower is $\min\{\Delta E_{ij}\}$, then the range is also $|\min\{\Delta E_{ij}\}|$. Then state 1 contains all edges with $\Delta E_{ij} \in [0; -\Delta]$, state 2 with $\Delta E_{ij} \in (-\Delta; -2\Delta]$, and so on. The lowest state contains mostly those interactions which are below the threshold value.

Of course, the energy spans $R^{(1)}$, $R^{(2)}$ of the two methods 1 and 2 are not necessarily equal. Nonetheless, if the number of states n is the same in both histograms, then diagonal elements ($k = l$) in $p_{kl}^{(x,y)}$ plots, as e.g. in Figs S7-S9, indicate that both methods produced equivalent edges, speaking relatively. In other words, the k-th energy state in range $R^{(1)}$ by one method is also the k-th in the range $R^{(2)}$ of the other method. On the other hand, off-diagonal elements ($k \neq l$) of $p_{kl}^{(x,y)}$ indicate systematic "error" of one method relative to the other.

One of the problems is the selection of a suitable value for n. The "elbow rule" can be applied to the dependence of MI, H, and C on n. This rule is often used if no other good justification can be found for the selection of a parameter (e.g. selecting a suitable k in k-mean clustering). It is based on finding a break in some trend, as is in this case the dependence shown in Figure S5. The break is at $n = 40$. Since the energy range of ΔE_{ij} values in Figure S5 is from 0 to -40 kcal mol^{-1}, the resolution of the states is roughly 1 kcal mol^{-1}. Hence, state 1 has all edges with $\Delta E_{ij} \in [0; -1]$ kcal mol^{-1}, state 2 with $\Delta E_{ij} \in (-1; -2]$, and so on.

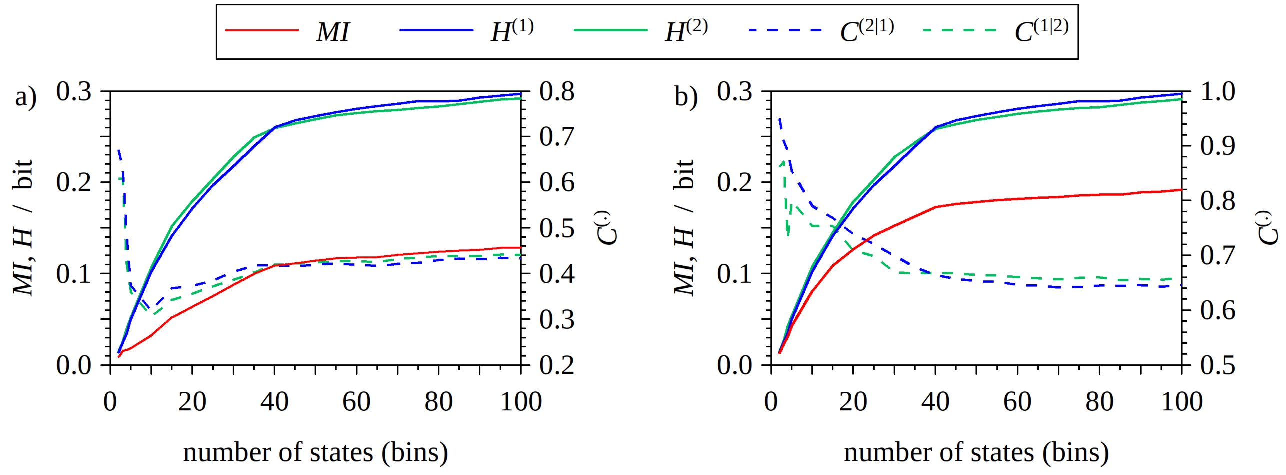

Figure S5: Dependence of MI, H and C on the number of states n used for histogram analysis. Part a) is for the case $G^{(1)}$ treated as FMO/solution/frag and $G^{(2)}$ as PA/solution/res. Part b) for $G^{(1)}$ treated as FMO/solution/frag and $G^{(2)}$ as PA/solution/frag.

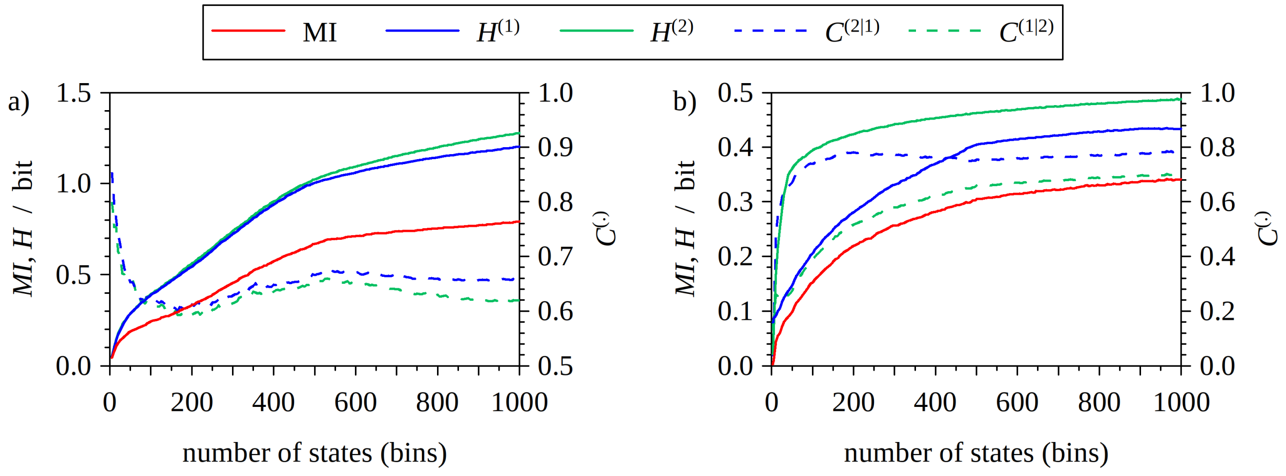

Figure S6: Dependence of MI, H and C on the number of states n used for histogram analysis. Both figures are for the case $G^{(1)}$ treated as FMO/solution/frag and $G^{(2)}$ as PA/solution/frag. Part a) depicts the case with a lowered acceptance threshold of $\Delta E_{ij} \leq 0$ kcal mol^{-1}. Part b) show the case if $\Delta E_{ij} \leq -1$ kcal mol^{-1}, but covalent bonds are accepted as edges.

The choice of assigning edges in G to residue pairs if $\Delta E_{ij} \leq -1$ kcal mol^{-1} is often used for PRNs. The physical reasoning is that relevant interactions are often stronger than this; see e.g. H-bond strength in the paper. Nevertheless, let us see how the acceptance of all attractive interactions changes our situation. Figure S6a depicts this case. The coefficients C do not reach an almost plateau until n is close to 550 states. The predictive value is

however not increased - it remains at best about 0.65 which is the same or slightly less than in Figure S5b.

The last issue to deal with is the acceptance of covalent bonds as edges in PRN graphs. In general these bonds are not used as edges, because the primary purpose of PRN models it to represent and analyse the complex networks of non-bonded residue-residue interactions. In the past this was in part motivated by the fact that FMO energies over covalent bonds are about one to two orders of magnitude larger that of the non-bonded interactions. See the interaction Met107:Asp108 in Table 1 in the main text and compare mainly PA/solution/res(frag) and FMO/solution/frag. This would result in a disproportional weight of these bonded edges in G compared to non-bonded edges and network algorithms based on shortest paths would produce results strongly favouring paths over the primary protein structure - the amino acid sequence, rendering the analysis of secondary, tertiary and quaternary structure buried as noise.[3] The new approach using PA analysis eliminates this problem to a large degree. Figure S6b depicts how asymmetric the information content becomes if we accept the covalent bonds as edges. $H^{(2)}$ for $G^{(2)}$ using the PA/solution/frag approach reaches "the elbow" at $n = 40$ (same as in Figure S5b) while $H^{(1)}$ for $G^{(1)}$ using the FMO/solution/frag does so at $n \sim 500$.

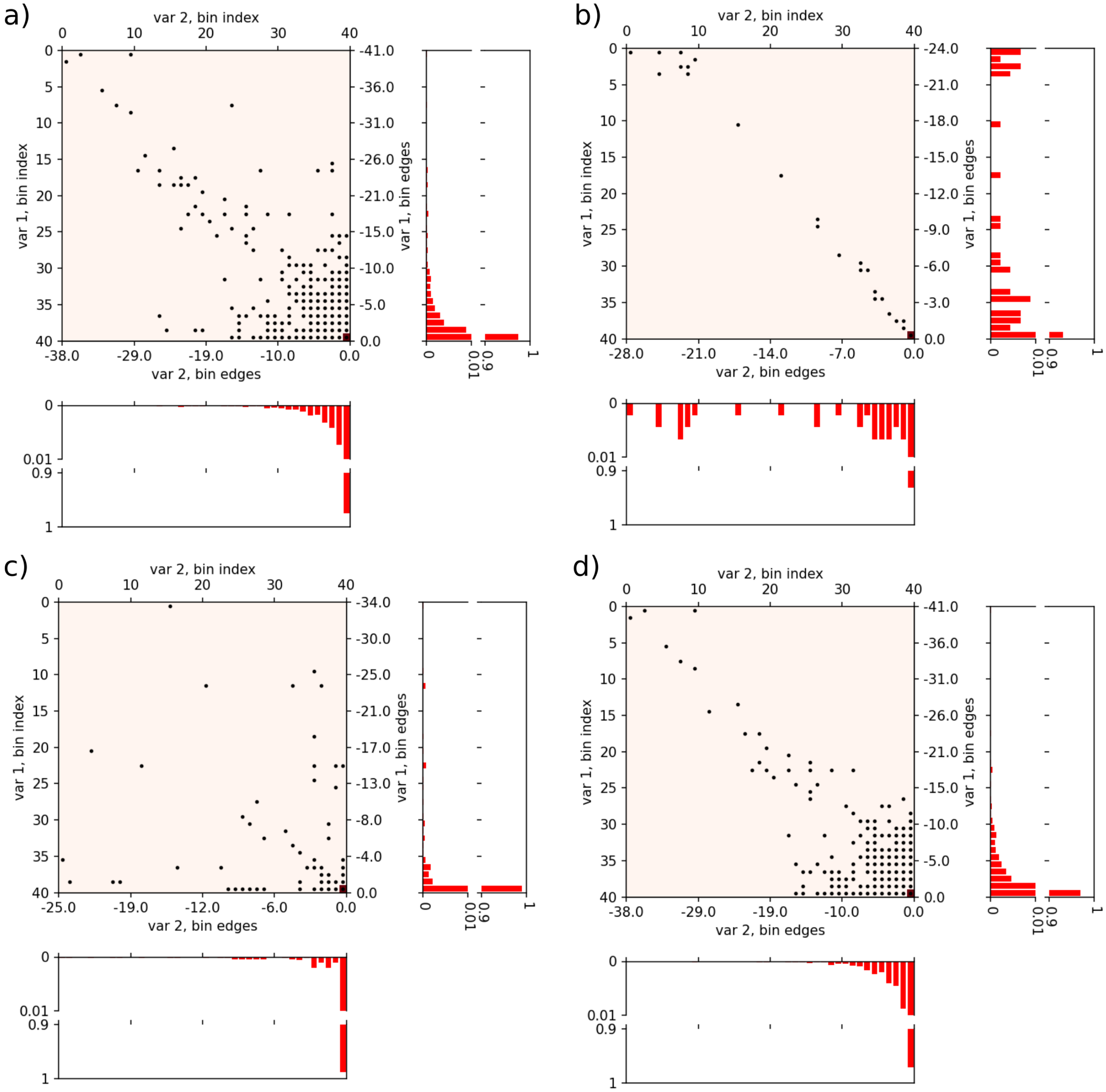

Figure S7: Probability distributions of edge states when vertices in $G^{(1)}$ are treated as FMO/solution/frag and in $G^{(2)}$ as PA/solution/res. There are $n = 40$ states along each axis. The vertical axes (variable 1) in the $2d$ joint probability distribution corresponds to states in $G^{(1)}$, the horizontal axes (var 2) to $G^{(2)}$. The axes indicate state/bin index as well as energy bin edges in kcal mol^{-1}. The same state/bin boundaries apply to the $1d$ marginal probability distributions shown as bar plots. Notice, the $2d$ plot does have a (linear) gradient colour map, but due to the extremely skewed distribution (see also the marginal $1d$ distributions) only the bottom right state is of noticeably different colour. Hence, dots mark all states when $p_{kl} \neq 0$. Part a) is for all monomer interactions; b) for nuc-nuc; c) for nuc-aa; d) for aa-aa only.

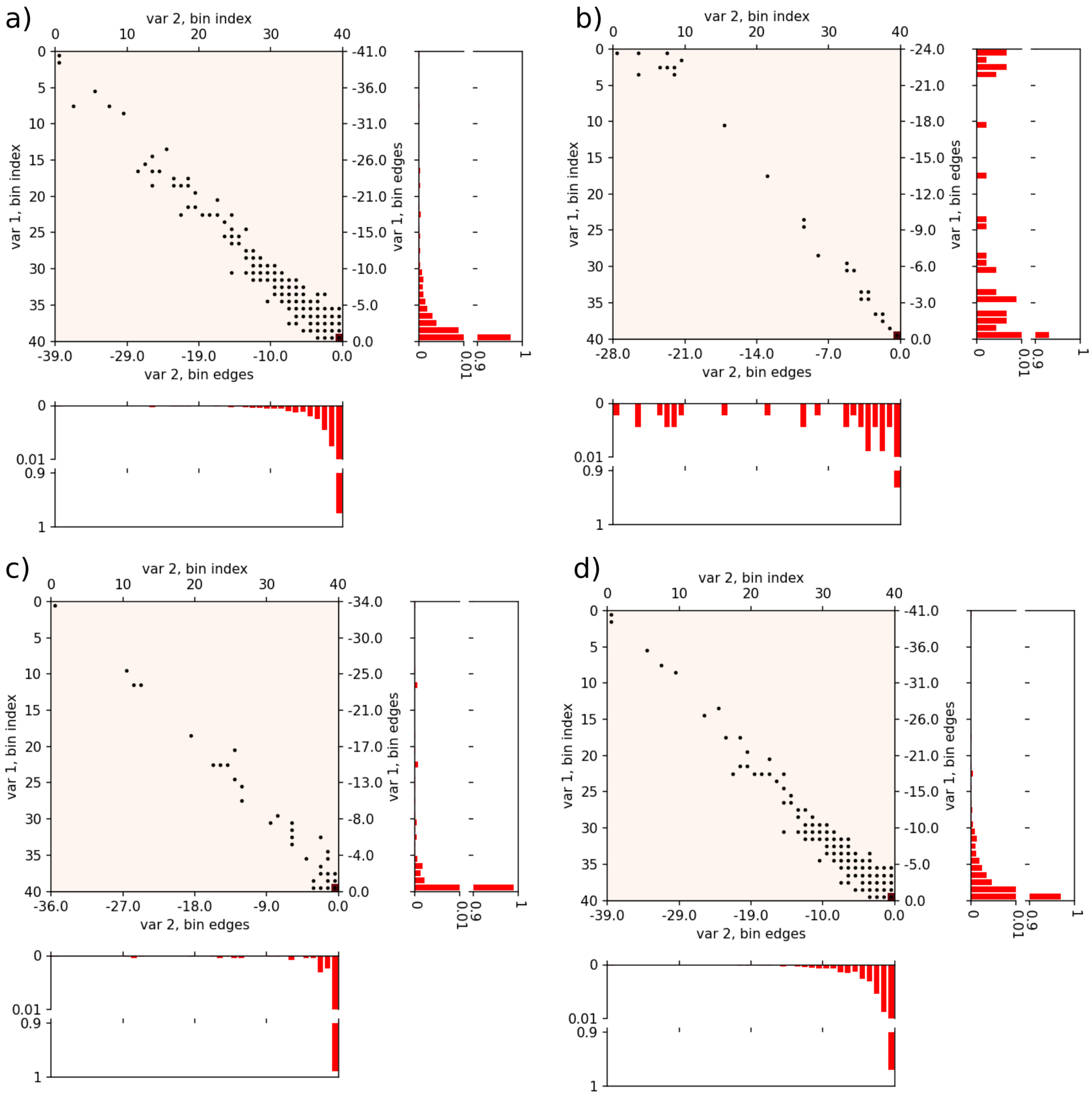

Figure S8: Probability distributions of edge states when vertices in $G^{(1)}$ are treated as FMO/solution/frag and in $G^{(2)}$ as PA/solution/frag. We use $n = 40$ states along each axis. The vertical axes (variable 1) in the $2d$ joint probability distribution corresponds to states in $G^{(1)}$, the horizontal axes (var 2) to $G^{(2)}$. The axes indicate state/bin index as well as energy bin edges in kcal mol^{-1}. The same state/bin boundaries apply to the $1d$ marginal probability distributions shown as bar plots. Notice, the $2d$ plot does have a (linear) gradient colour map, but due to the extremely skewed distribution (see also the marginal $1d$ distributions) only the bottom right state is of noticeably different colour. Hence, dots mark all states when $p_{kl} \neq 0$. Part a) is for all monomer interactions; b) for nuc-nuc; c) for nuc-aa; d) for aa-aa only.

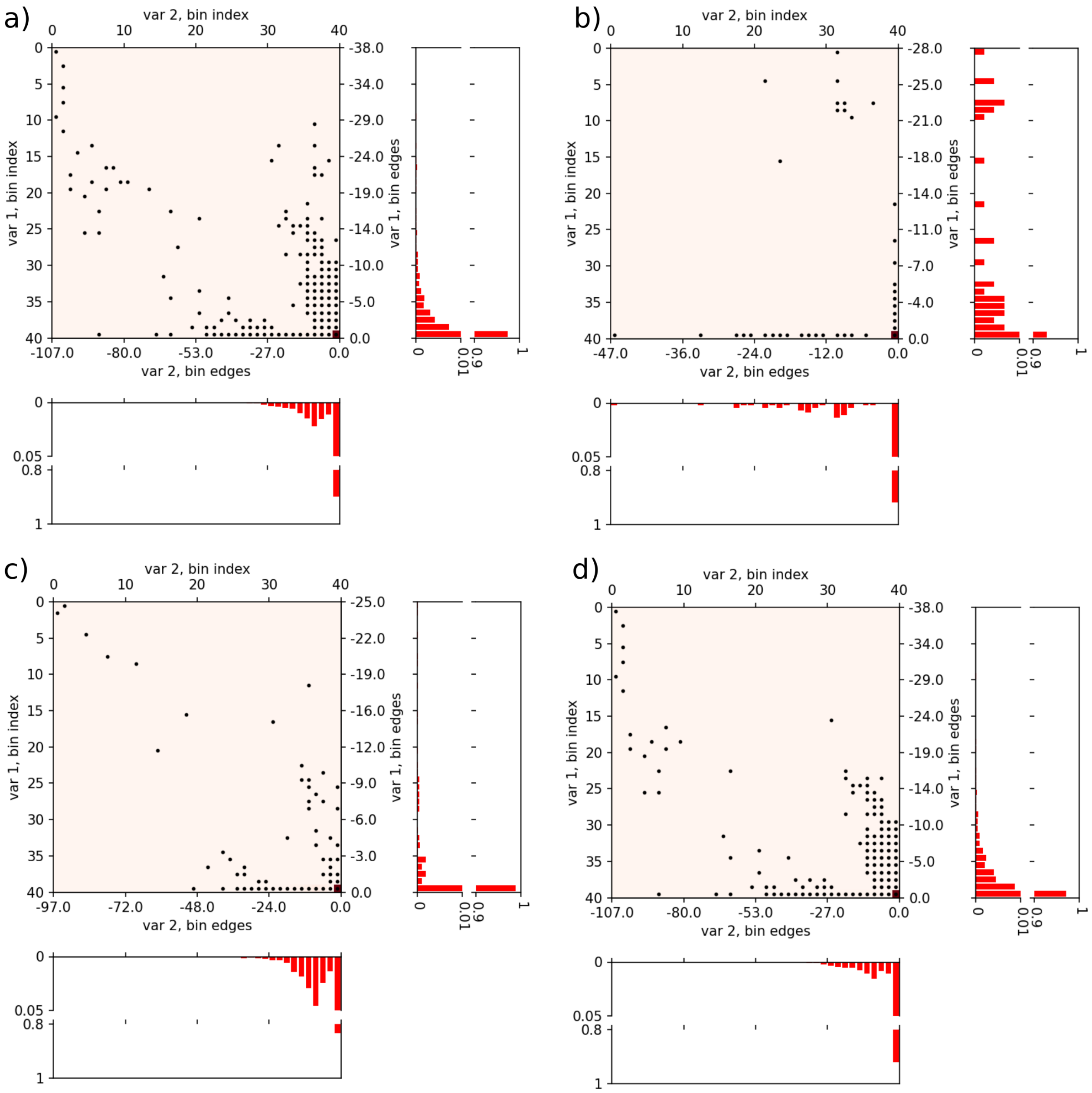

Figure S9: Probability distributions of edge states when vertices in $G^{(1)}$ are treated as PA/solution/res and in $G^{(2)}$ as MM/solute/res. There are $n = 40$ states along each axis. The vertical axes (variable 1) in the $2d$ joint probability distribution corresponds to states in $G^{(1)}$, the horizontal axes (var 2) to $G^{(2)}$. The axes indicate state/bin index as well as energy bin edges in kcal mol^{-1}. The same state/bin boundaries apply to the $1d$ marginal probability distributions shown as bar plots. Notice, the $2d$ plot does have a (linear) gradient colour map, but due to the extremely skewed distribution (see also the marginal $1d$ distributions) only the bottom right state is of noticeably different colour. Hence, dots mark all states when $p_{kl} \neq 0$. Part a) is for all monomer interactions; b) for nuc-nuc; c) for nuc-aa; d) for aa-aa only.

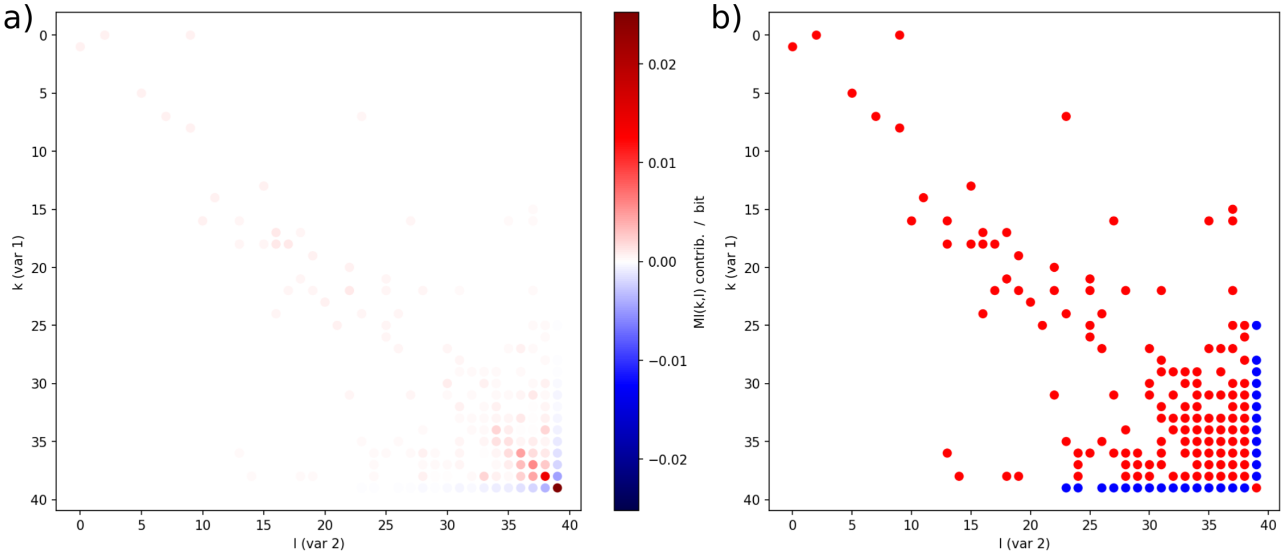

Figure S10: Contribution of states in the probability distribution of edge states when vertices in $G^{(1)}$ are treated as FMO/solution/frag and in $G^{(2)}$ as PA/solution/res. There are $n = 40$ states along each axis. The vertical axes (variable 1) in the $2d$ joint probability distribution corresponds to states in $G^{(1)}$, the horizontal axes (var 2) to $G^{(2)}$. The axes indicate state/bin index. The plots correspond to Figure S7a. Part a) shows the magnitude of contribution of each (k, l) state to MI (the kernel in the sum in eq. 2). Red color means a positive contribution or raising MI, blue color depicts a negative one or lowering MI. Part b) shows the same, but the coloring is discrete (magnitude not reflected).

Table S1: Average graph density for various methods of calculating edge energies (PIEs) when $E_{\text{lim}} = -1$ kcal mol^{-1}.[a]

density	FMO/solution/frag	PA/solution/res	PA/solution/frag	MM/solute/res
$D(G)$	0.02562	0.02515	0.02532	0.12224

[a] Graph density is the ratio of number of edges vs maximum theoretical number of edges (for a complete graph the density is 1). So if N is the number of nodes, then density $D(G) = 2|e|/(N(N-1))$, where $|e|$ is the number of edges in G.

References

(1) Bossomaier, T.; Barnett, L.; Harré, M.; Lizier, J. T. *An Introduction to Transfer Entropy: Information Flow in Complex Systems*; Springer International Publishing, 2016.

(2) Ben-Naim, A. *A Farewell to Entropy: Statistical Thermodynamics Based on Information*; World Scientific, 2008.

(3) Sladek, V.; Tokiwa, H.; Hitoshi, S.; Yasuteru, S. Protein Residue Networks from Energetic and Geometric Data: Are They Identical? *J. Chem. Theory Comput.* **2018**, *14*, 6623–6631.
